# The effects of heavy smoking on oxidative stress, inflammatory biomarkers, vascular dysfunction, and hematological indices

**DOI:** 10.1038/s41598-025-03075-8

**Published:** 2025-05-25

**Authors:** Zhikal Omar Khudhur, Shukur Wasman Smail, Harem Khdir Awla, Gasheen Bakhtiyar Ahmed, Yara Omar Khdhir, Kawa Amin, Christer Janson

**Affiliations:** 1 Biology Education Department, Tishk International University, Erbil, Iraq; 2 Department of Biology, College of Science, Salahaddin University-Erbil, Erbil, Kurdistan Region Iraq; 3 College of Pharmacy, Cihan University-Erbil, Erbil, Kurdistan Region Iraq; 4 Radiological imaging technology Department, College of Health Technology, Cihan University-Erbil, Erbil, Kurdistan Region Iraq; 5 Department of Medicine, Microbiology/Immunology, College of Medicine, University of Sulaimani, Sulaymaniyah, Iraq; 6 Department of Medical Science, Respiratory, Allergy and Sleep Research, Uppsala University and University Hospital, Uppsala, Sweden

**Keywords:** Smoking, Endothelin-1, Inflammatory biomarkers, Hematological indices, And vascular dysfunction, Respiration, Chemokines, Biochemistry, Immunology

## Abstract

Smoking remains a major health issue worldwide, causing various diseases. This research examines the effects of heavy smoking on oxidative stress, inflammatory biomarkers, blood vascular health, and hematological parameters. A study conducted at Par Private Hospital in Erbil, Iraq, from April to May 2024 examined 104 male heavy smokers and 94 healthy male nonsmokers. Blood and biopsy samples were collected from both groups. The groups had similar age and body mass index, reducing the chances of confounding factors. Smokers showed significantly higher levels of oxidative stress markers, including malondialdehyde (*p* = 0.002) and nitric oxide (*p* = 0.002), and lower superoxide dismutase activity (*p* < 0.0001) compared to controls. Smokers displayed significantly elevated levels of serum endothelin-1 and interleukin-8 (*p* < 0.0001). Immunohistochemical analysis showed a significant rise in myeloperoxidase-positive cells and eosinophilic granule 2-positive eosinophils among smokers, indicating heightened neutrophil and eosinophil activity. Smokers showed elevated neutrophil counts in hematological analysis, while lymphocyte counts and neutrophil-to-lymphocyte ratio remained unchanged. Oxidative stress parameters, endothelin-1 levels, inflammatory biomarkers, and hematological indices are all significantly influenced by heavy smoking, reflecting systemic inflammation and vascular dysfunction. Public health initiatives are urgently required to address the harmful health consequences of smoking, as indicated by noteworthy changes in inflammation and oxidative stress biomarkers. The study offers important information on the physical effects of long-term heavy smoking, enhancing our understanding of the health hazards associated with smoking.

## Introduction

Smoking remains a significant burden on the global economy and public health. Over eight million deaths occur globally, with seven million resulting from direct tobacco use and 1.3 million attributed to secondhand smoke exposure^1,2^. Heavy smokers are individuals who are accustomed to smoking over twenty cigarettes per day. The majority of smoking occurs in impoverished nations^3^. Smoking leads to the production of carbon monoxide, which binds to hemoglobin and results in hypoxia. Smoking is a risk factor for inflammation, endothelial dysfunction, cancer, stroke, heart attack, respiratory diseases, and inflammatory diseases^4,5^.

The harmful constituents of cigarette smoke, such as nicotine and numerous other toxic chemicals, contribute to increased oxidative stress and endothelial dysfunction^6^. with elevated levels of biomarkers like 8-isoprostaglandin F2α (8-isoPGF2α) and soluble NOX2-derived peptide (sNOX2-dp). This oxidative stress can impair endothelial function and promote inflammation, which are key mechanisms underlying smoking-related cardiovascular disease^7^. Malondialdehyde (MDA), a marker of lipid peroxidation, is found at significantly higher levels in the blood of heavy smokers than nonsmokers, particularly in those with respiratory diseases like asthma and COPD^8^. Smoking impairs nitric oxide (NO) bioavailability and endothelial function due to oxidative stress, which reduces endothelial nitric oxide synthase (eNOS) production and activity. Additionally, heavy smoking decreases antioxidant defenses, including the activity of enzymes like superoxide dismutase (SOD), further contributing to oxidative stress^7^.

In addition to oxidative stress, smoking has also been shown to impact other physiological parameters. For example, smoking can increase levels of the vasoconstrictor endothelin 1, a potent vasoconstrictor^9,10^. Endothelin-1 is primarily produced by endothelial cells and is crucial in regulating vascular tone and homeostasis. Smoking has been found to increase endothelin-1 levels, which contributes to endothelial dysfunction and vascular damage^11^. Smoking can also affect complete blood count, with studies reporting changes in hematological indices like hemoglobin, hematocrit, and platelet counts in smokers compared to nonsmokers^12^. On the other hand, cigarette smoking has severe adverse on hematological parameters, that lead to significant increases in red blood cells (RBC), Hb, and white blood cells, which may be associated with a greater risk of developing serious health conditions^13^.

Interleukin (IL)-8, eosinophils, and myeloperoxidase (MPO) are among the inflammatory markers that are significantly influenced by smoking. The presence of high levels of IL-8, an important pro-inflammatory cytokine, is consistently observed in smokers, leading to increased airway inflammation and playing a role in chronic obstructive pulmonary disease (COPD)^14^.

Additionally, biopsy-based assessments of MPO-positive cells (Neutrophil and monocyte and activated eosinophils (eosinophilic granule 2 (EG2)) positive cells in bronchial tissue provide insights into the local immune response and structural changes characteristic of allergic asthma. Furthermore, heightened allergic and inflammatory responses are observed in symptomatic smokers, indicating eosinophil activation and increased eosinophil cationic protein (ECP) levels^15^. Smokers show elevated levels of MPO, an enzyme found in neutrophils and monocytes, which indicates a heightened inflammatory response driven by neutrophils^16^. Smoking-induced chronic inflammation is the main factor behind the elevated inflammatory markers and the development of smoking-related respiratory diseases.

While several studies have explored the effects of smoking on oxidative stress or inflammatory pathways individually, relatively few have simultaneously assessed biochemical, hematological, and immunohistochemical markers in the same cohort. Moreover, many existing studies focus on specific populations or limited biomarker profiles. Our study aims to address this gap by providing a comprehensive analysis of smoking-related changes in oxidative stress, inflammatory biomarkers, vascular dysfunction, and immune cell infiltration, thereby offering a more integrative understanding of smoking’s systemic effects.

Therefore, this study aims to provide a comprehensive evaluation of the effects of heavy smoking on oxidative stress markers, inflammatory cytokines, vascular dysfunction indicators, and hematological parameters in comparison to healthy nonsmokers. By analyzing both blood and bronchial biopsy samples, we aim to contribute a broader and more integrated perspective on smoking-related systemic alterations.

## Materials and methods

### Study design

The study took place at Par Private Hospital in Erbil-Iraq from April 1st to May 20th, 2024. The study included into involved two groups: heavy smokers and healthy nonsmokers. The heavy smoker group consisted of 104 males who smoked more than 20 cigarettes daily for a minimum duration of 5 years. In contrast, the healthy nonsmokers group included 94 individuals with no history of smoking and minimal exposure to secondhand smoke. Only male smokers were included to minimize the potential confounding effects of sex hormones and gender-related physiological differences that could influence oxidative stress markers, immune responses, and hematological parameters. Estrogen, for instance, has been shown to exert antioxidant effects and modulate immune function, potentially altering biomarker expression and making direct comparisons between male and female smokers less reliable^17^.

Furthermore, in Iraq and many neighboring countries, the prevalence of female smokers remains significantly low due to strong cultural, religious, and social norms that discourage tobacco use among women. Several regional surveys and public health reports have confirmed that female smoking rates are disproportionately lower than male rates, often below 5% ^18^. The societal stigma and religious disapproval associated with smoking among women in Iraq make it challenging to recruit a representative sample size for meaningful statistical analysis. As this study is based on original research and is not a systematic review, no risk of bias tool or PRISMA framework was applied, rather it is based on STROBE (strengthening the reporting of observational studies in epidemiology) guidelines for the reporting of observational studies. However, efforts were made to minimize bias by matching participants for age and BMI and using standardized procedures for sample collection and laboratory analysis.

**Inclusion criteria**:

1. Participants must be male.
2. Participants must be of Kurdish ethnicity.
3. Heavy smokers must smoke more than 20 cigarettes per day over five years.
4. Healthy nonsmokers must have no history of smoking and minimal exposure to secondhand smoke.
5. Participants must be aged between 18 and 60 years.
6. Participants must provide informed consent to participate in the study.

**Exclusion criteria**:

1. Individuals with a history of chronic diseases such as diabetes, hypertension, or cardiovascular diseases.
2. Individuals with any acute or chronic respiratory diseases not related to smoking.
3. Individuals currently taking any medication that could affect oxidative stress or endothelial function.
4. Individuals with a history of substance abuse other than tobacco.
5. Individuals who have undergone any major surgical procedure in the past six months.
6. Individuals with any known inflammatory or autoimmune conditions.
7. Individuals who are not willing or able to provide informed consent.

### Sample collection and laboratory tests

This study was approved by the Human Ethics Committee of the College of Science, Salahaddin University-Erbil, under approval number R0234, 011, dated March 2nd, 2023. The study was conducted in accordance with the Declaration of Helsinki and all relevant institutional and national guidelines and regulations. Written informed consent was obtained from all participants prior to sample collection and testing. Participants were fully informed about the purpose of the study, the procedures involved, and any potential risks or benefits. Blood samples were collected from everyone under standardized conditions. Only 35 individuals (16 controls and 19 smokers) underwent biopsies for IHC staining. The blood was obtained and put in serum separation tubes (SSTs) and EDTA tubes in preparation for serological testing and hematological testing, respectively. Hematological parameters were measured by a hematological analyzer (Sysmex XT-2000i, Japan). NLR is obtained by dividing neutrophil counts by lymphocyte counts. Blood pressure was measured using a mercury sphygmomanometer (Riester Diplomat^®^, Rudolf Riester GmbH, Germany) and a standard stethoscope (Littmann Classic III™, 3 M, USA) following standard procedures. Measurements were taken from the left arm of each seated participant after at least 5 min of rest, with an average of two readings recorded. Body mass index (BMI) was calculated as body weight (kg) divided by body height (m) squared.

The SST was left undisturbed for half an hour to allow the blood to clot. A temperature of -70 degrees Celsius was maintained to store the serum samples. At Par-hospital in Erbil-City, Iraq, a respiratory pulmonologist performed a bronchial biopsy using fiber bronchoscopy. The par hospital database was utilized to record data for each patient and complete the questionnaire for this study. The questionnaire includes information such as age, BMI, systolic blood pressure (SBP), medical history, clinical tests, and asthma severity scores.

### Serum malondialdehyde and nitric oxide determination

Malondialdehyde (MDA) levels were determined using a commercial MDA assay kit (Navand Salamat Company, Urmia, Iran), with sample absorbance measured at 523 nm using a spectrophotometer (Thermo Fisher Scientific, Waltham, MA), and the results expressed in nmol/mg of protein. On the other hand, NO levels were assessed using an NO assay kit (Navand Salamat Company, Urmia, Iran). Sample absorbance was read at 570 nm, and NO concentration was reported as nmol/mg protein. These kits were also used by other researchers, such as Moradi, et al.^19^, Zolfaghari, et al.^20^ and Shareef, et al.^21^.

### Measurement of serum endothelin-1 and IL-8

Serum endothelin-1 and IL-8 levels were measured using an ELISA kit from RD Systems, Inc., Minneapolis, MN, USA, following the manufacturer’s protocols.

### The procedure of immunohistochemistry

The polyclonal antibodies for anti-EG2 and anti-MPO were employed to identify activated eosinophils and polymorphonuclear leukocytes (neutrophils and monocytes), respectively. The antibodies were purchased from Antibodies, GmbH, a company based in Germany, with a concentration of 0.5 µg/ml. The biopsy, which had been thawed, was treated with a concentrated Ortho Permeafix solution (Orthod Diagnostics, USA) for approximately 40 min at room temperature. Later, it was subjected to suitable polyclonal antibodies for each marker for approximately 60 min in a chamber with humidity control. Subsequently, it was washed with PBS (pH 7.2) for around 3 min. The section was then incubated at room temperature for 45 min with a conjugated antibody (goat anti-rabbit antibody with HRP purchased from DAKO, USA (code: K8002).

Following the incubation period with the secondary antibody, the tissue sections (already mounted on slides) were rinsed with PBS (pH 7.2) for approximately 5 min. Subsequently, the chromogenic substrate (DAB + Chromogen; DAKO, USA, code: K8002) was applied directly to the tissue sections on the slides and incubated for 10 min at room temperature. The slides were then rinsed with distilled water to remove excess chromogen, counterstained with hematoxylin for 10 min, and washed again with tap water. Afterwards, a mounting medium (DAKO, USA) was applied, and coverslips were placed onto the slides. The slides were air-dried and examined under a Leica light microscope (Germany) at magnifications of 20× and 40×. A digital camera (DC 300 F) attached to the microscope captured images, and Qwin v2.7 software was used to quantify positive cells per square millimeter.

### Statistical analysis

GraphPad Prism 6.0 was used for statistical analysis and data representation. The data passing the normality test (DeAgostino, Shapiro, and Kolmogorov) undergo parametric analysis, while the ones failing it undergo non-parametric analysis. The parameters of heavy smokers were compared to those of the control group using an independent t-test (for parametric data) and a Mann-Whitney test (for non-parametric data). The parametric data included the mean and standard error of the mean (SEM), while the non-parametric data included the median and 25-75% interquartile range (IQR). The Spearman test was employed to assess the correlation between variables. A p-value below 0.05 was deemed statistically significant.

## Result

The average age of heavy smokers was 26.13 ± 0.614 years, and that of the control group was 26.85 ± 0.953 years (*p* = 0.533). Similarly, there was no significant difference in BMI between the control group (25.31 ± 0.578) and smokers (24.73 ± 0.450) (*p* = 0.436), as shown in Table 1.

**Table 1:** Comparison of laboratory parameters between smokers and healthy controls

Parameters	Control (Mean ± SEM) *n* = 94	Smoker (Mean ± SEM) *n* = 104	*P*-value
n	94	104	–
Age (years)	26.85 ± 0.953	26.13 ± 0.614	0.533
BMI (Kg/m^2^)	25.31 ± 0.578	24.73 ± 0.450	0.436
RBC (10^12^/l)	5.081 ± 0.482	6.281 ± 0.56	0.104
Hb (g/dl)	14.14 ± 0.198	16.93 ± 0.217	< 0.0001
WBC (10^9^/l)	6.411 ± 0.144	9.378 ± 0.399	< 0.0001
Platelet (10^3^/µl)	277.3 ± 7.488	218.6 ± 9.065	< 0.0001
Lymphocyte (10^9^/l)	1.921 ± 0.067	2.051 ± 0.056	0.143
Neutrophil (10^9^/l)	3.996 ± 0.116	4.620 ± 0.152	0.001
NLR	2.362 ± 0.122	2.318 ± 0.076	0.759
SBP (mmHg)	120.1 ± 1.157	121.9 ± 0.859	0.204
MDA (µmol/L)	5.294 ± 0.274	6.324 ± 0.167	0.002
NO (µmol/L)	24.66 ± 0.508	27.43 ± 0.726	0.002
SOD activity (U/ml)	15.03 ± 0.164	13.88 ± 0.142	< 0.0001
Endothelin-1 (pg/ml)	30.05 ± 1.931	150.2 ± 26.90	< 0.0001
IL-8 (pg/ml)	41.56 ± 0.593	54.45 ± 1.207	< 0.0001

The independent t-test was utilized for comparison. A p-value below 0.05 is considered statistically significant. The data is represented by Mean ± standard error of mean (SEM)

*BMI* body mass index, *Hb* hemoglobin, *IL* interleukin, *MDA* malondialdehyde, *n* sample size, *NLR* Neutrophil-to- lymphocyte ratio, *NO* nitric oxide, *RBC* red blood cell, SBP systolic blood pressure, SOD superoxide dismutase activity, *WBC* white blood cell

### Comparing systolic blood pressure in the control group and smokers

Systolic blood pressure (SBP) was similar between the two groups, with controls at 120.1 ± 1.157 mmHg and smokers at 121.9 ± 0.859 mmHg (*p* = 0.204).

### Variations in hematological indices between control and smokers

The mean lymphocyte count was 1.921 ± 0.067 (10^9^/l) in controls and 2.051 ± 0.056 (10^9^/l) in smokers, showing no significant difference (*p* = 0.143). However, the mean neutrophil count was significantly higher in smokers (4.620 ± 0.152 (10^9^/l)) compared to controls (3.996 ± 0.116 (10^9^/l)), with a p-value of 0.001. The neutrophil-to-lymphocyte ratio (NLR) was 2.362 ± 0.122 in controls and 2.318 ± 0.076 in smokers, with no significant difference (*p* = 0.759). This elevated neutrophil count in smokers suggests an enhanced systemic inflammatory response.

**Fig. 1:**
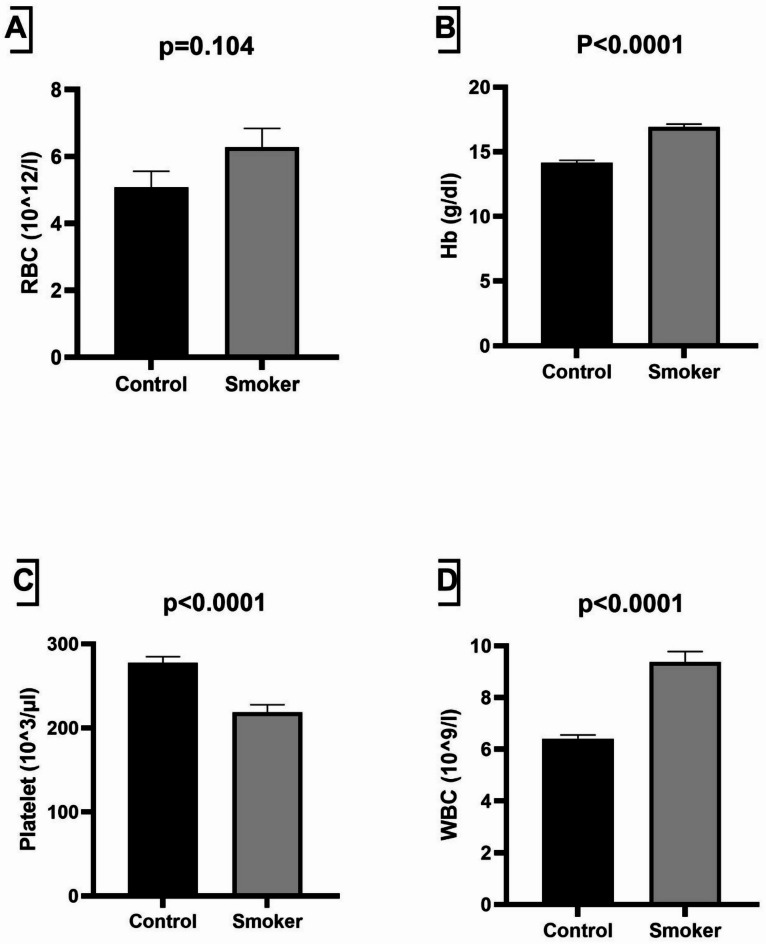
Analyzing the differences in some hematological parameters between healthy control and smokers. The independent t-test was utilized for comparison of **A** RBC, **B** Hb, **C** platelet, and **D** WBC. A p-value below 0.05 is considered statistically significant. The data is represented by Mean ± standard error of mean (SEM). *Hb* hemoglobin, *RBC* red blood cell, *WBC* white blood cell

The Hb levels between smokers and healthy controls show no significant differences (p-value = 0.104). Table 1; Fig. 1 show a significant increase (p-value < 0.000) in RBC and WBC, but a significant decrease (p-value < 0.000) in platelets between smokers and healthy controls. These changes in hematological parameters suggest that heavy smoking may lead to blood viscosity alterations and enhanced inflammatory responses, which are risk factors for thrombosis.

### Differences in oxidative stress markers and serum endothelin-1 levels between nonsmokers and smokers

**Fig. 2:**
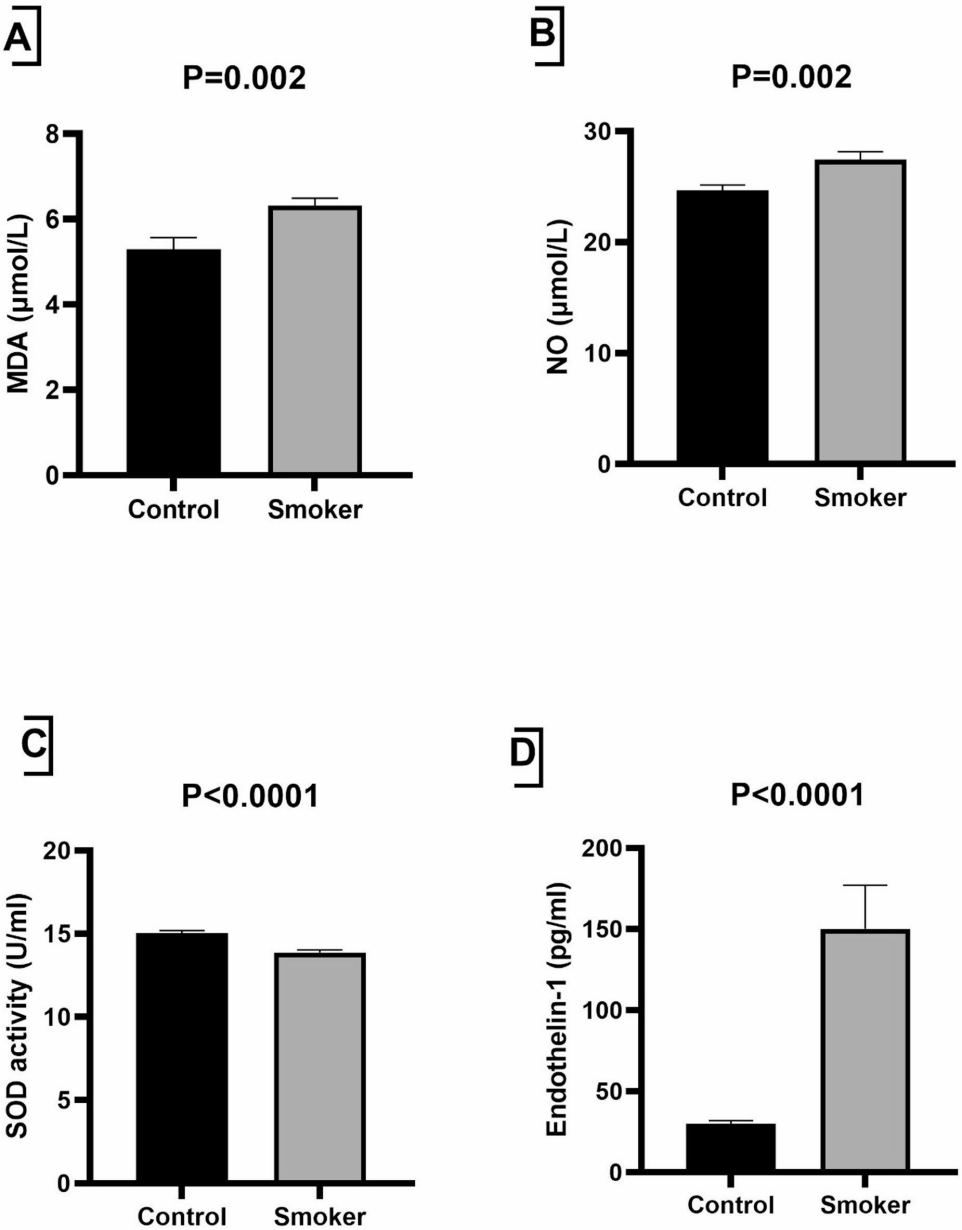
Analyzing the differences in oxidative stress markers and endothelin-1 between healthy control and smokers. The independent t-test was utilized for comparison of **A** MDA, **B** NO, **C** SOD activity, and **D** endothelin-1. A p-value below 0.05 is considered statistically significant. The data is represented by Mean. *MDA* malondialdehyde, *NO* nitric oxide, *SOD* superoxide dismutase activity

Malondialdehyde (MDA) levels were significantly higher in smokers (6.324 ± 0.167 µmol/L) compared to controls (5.294 ± 0.274 µmol/L), with a p-value of 0.002. NO levels were also significantly elevated in smokers (27.43 ± 0.726 µmol/L) compared to controls (24.66 ± 0.508 µmol/L), with a p-value of 0.002. SOD activity was significantly lower in smokers (13.88 ± 0.142 U/ml) compared to controls (15.03 ± 0.164 U/ml), with a p-value of < 0.0001. Endothelin-1 levels were markedly higher in smokers (150.2 ± 26.90 pg/ml) compared to controls (30.05 ± 1.931 pg/ml), with a p-value of < 0.0001 (Fig. 2).

### Analyzing the difference in IL-8 between control and smoker groups

**Fig. 3:**
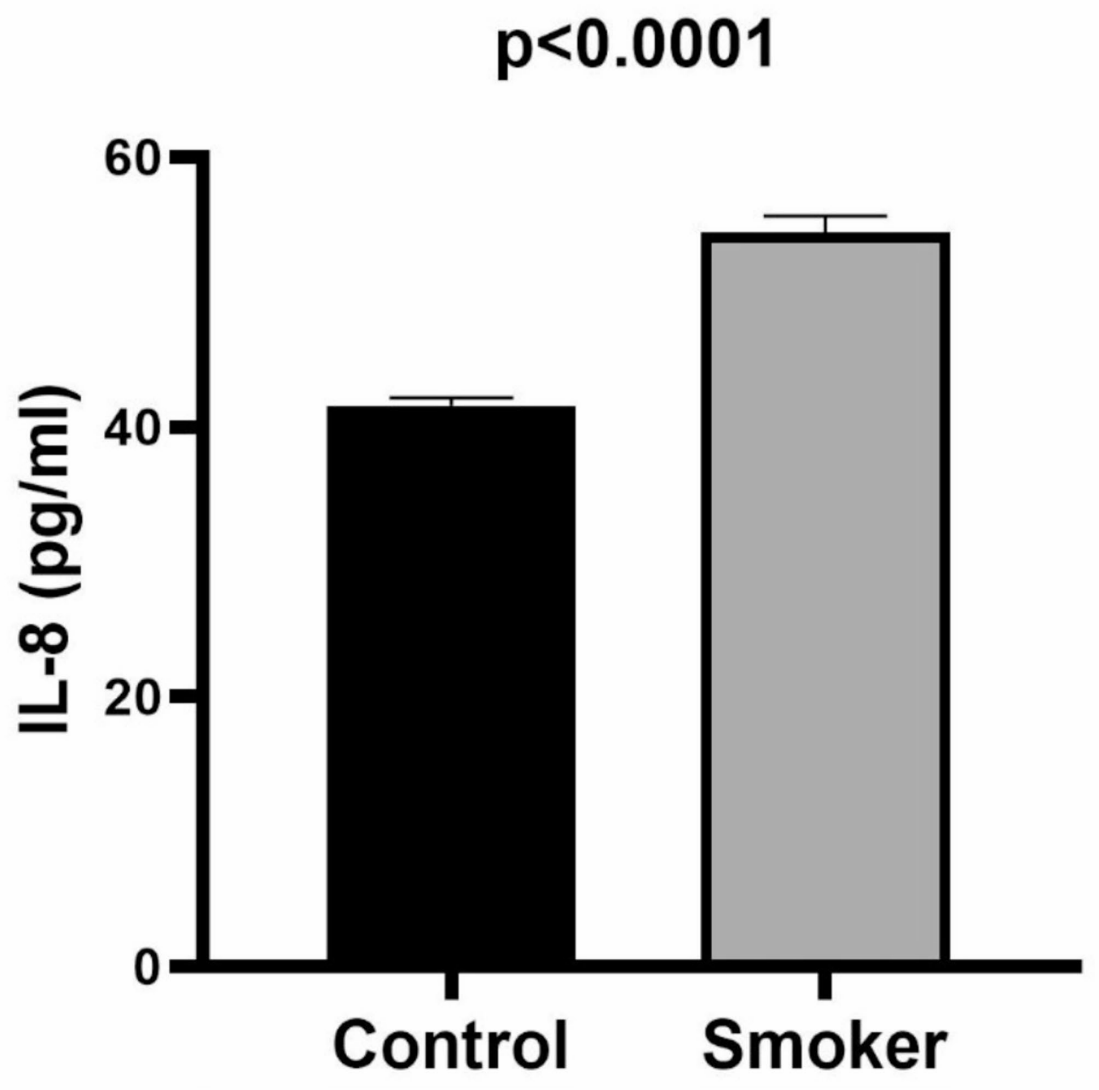
Examining the difference in serum IL-8 levels between smokers and healthy individuals. The independent t-test was utilized for comparison. The data is represented by Mean + SEM. *IL* interleukin

Smokers exhibit a significantly higher mean IL-8 level (54.45 ± 1.207 pg/ml) compared to the control group (41.56 ± 0.593 pg/ml) (Table 1; Figure 3).

### Comparison of immunohistochemistry biomarkers between control and smoker

**Fig. 4:**
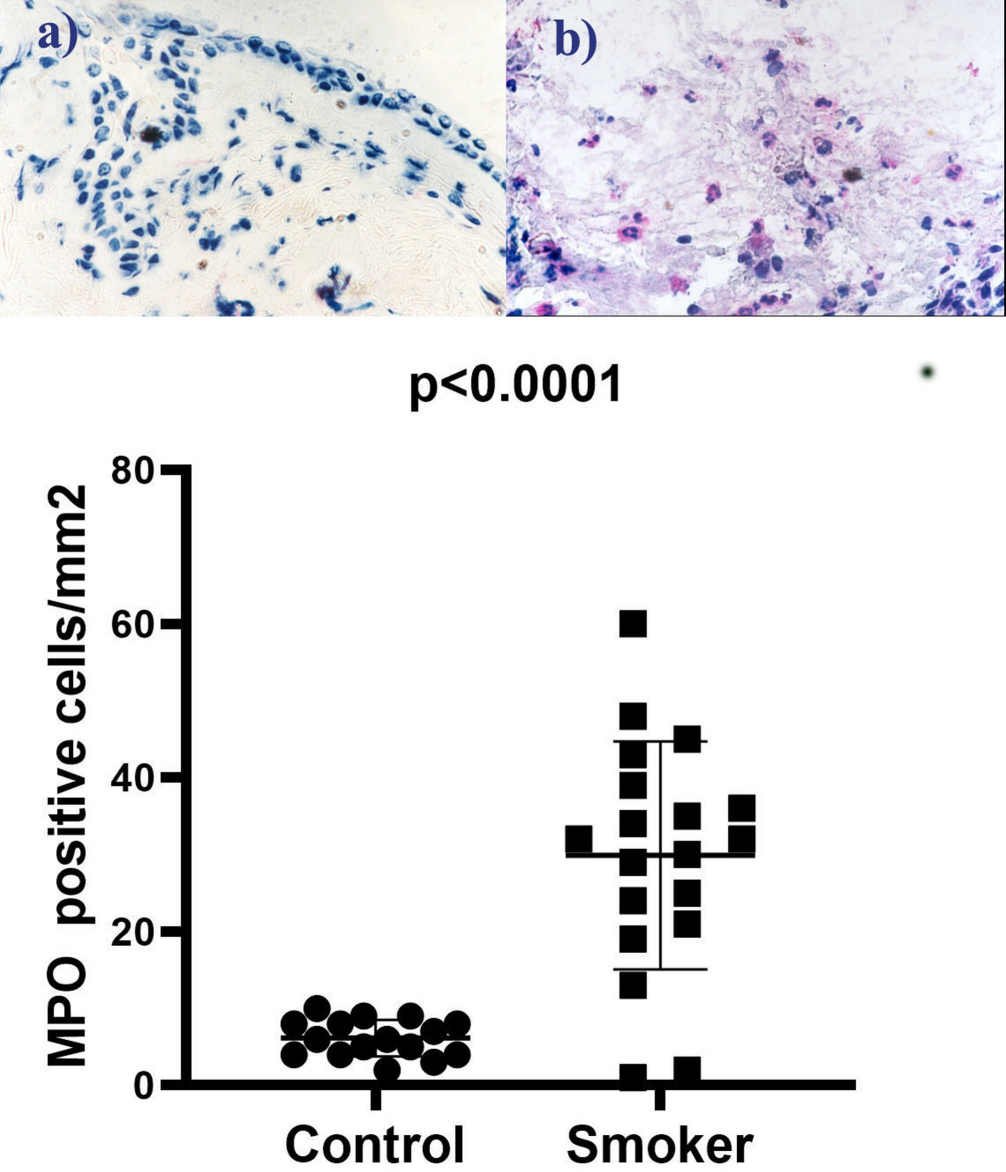
MPO positive cells with smoker (**b**) and healthy control (**a**). In individuals with smoker (**b**), there is a higher presence of MPO positive cells compared to healthy controls (**a**). MPO is an enzyme found in neutrophils and eosinophils, which are immune cells involved in the inflammatory response. In smokers, exposure to allergens triggers an exaggerated immune response, leading to airway inflammation and increased MPO activity. This heightened MPO activity contributes to tissue damage and worsens the symptoms of asthma, such as wheezing, coughing, and shortness of breath. In contrast, healthy individuals have a lower number of MPO positive cells, indicating a well-regulated immune response and absence of significant airway inflammation. The presentation of the data included the use of the median and interquartile range (IQR). The comparison between the two groups was done using the Mann-Whitney test. Statistical significance was attributed to p-values below 0.05. *MPO* myeloperoxidase

**Fig. 5:**
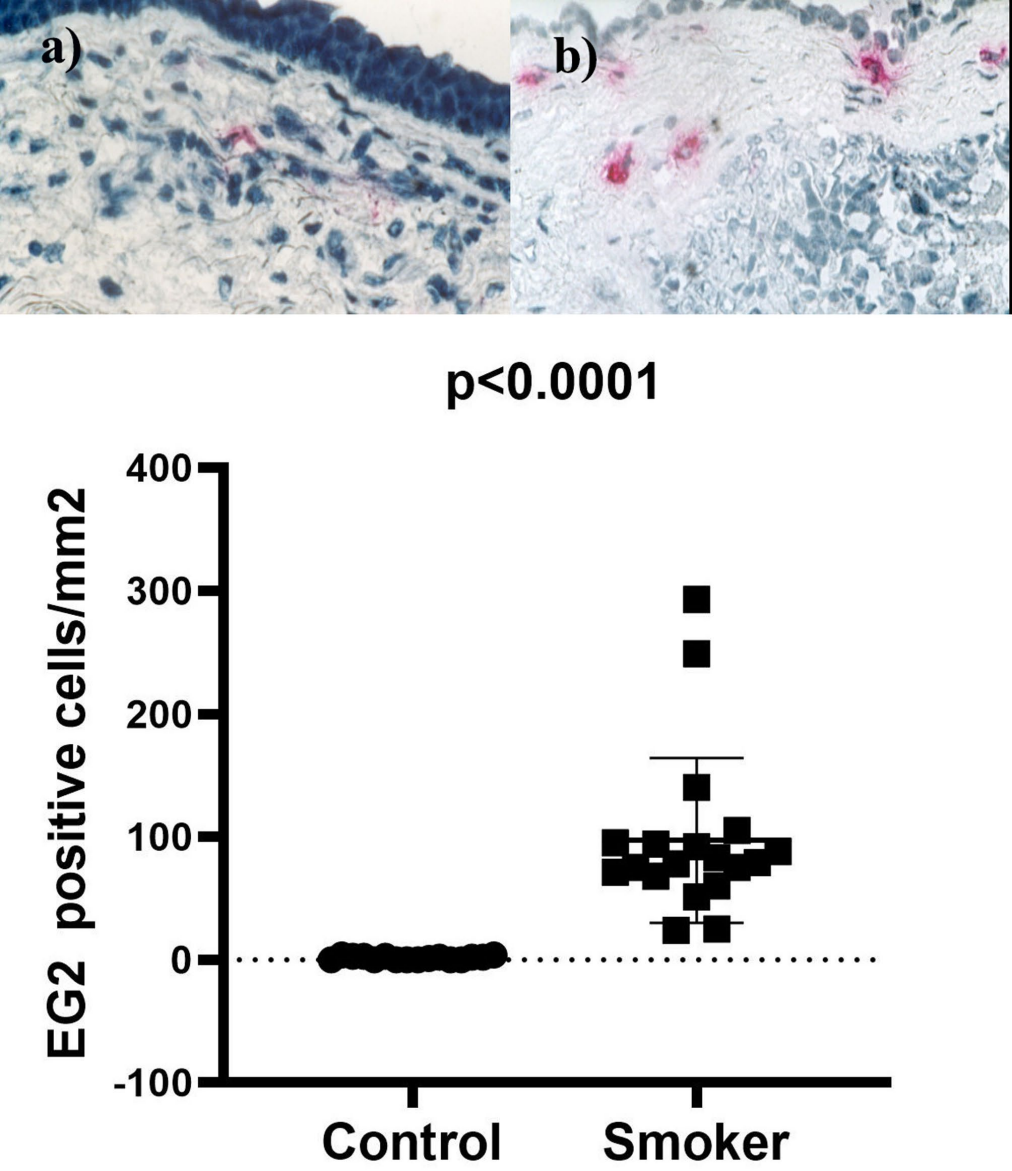
EG2 positive cells with smoker (**b**) and healthy control (**a**). In allergic asthma, EG2 positive cells play a significant role in the immune response. These cells, predominantly eosinophils, are involved in the release of inflammatory mediators (ECP) that contribute to the characteristic airway inflammation and bronchoconstriction seen in smoker. In individuals with smoking (**b**), there is an increased presence of EG2 positive cells compared to healthy controls (**a**). This elevation in EG2 positive cells is a hallmark of allergic inflammation and reflects the heightened immune response in individuals with smoking. In contrast, healthy individuals (**a**) have lower levels of EG2 positive cells in their airways, indicating a balanced immune system and absence of ongoing allergic inflammation. The presentation of the data included the use of the median and interquartile range (IQR). The comparison between the two groups was done using the Mann-Whitney test. Statistical significance was attributed to p-values below 0.05. *EG2* eosinophilic granule 2

There is a statistically significant difference in the median value of MPO-positive cells between smokers (32) and the control group (6), with a p-value < 0.0001, implying a substantial increase in polymorphonuclear leukocytes in smokers. Activated eosinophils show a statistically significant increase in smokers, as the median value of EG2 positive cells is significantly higher (79) compared to the control group (1.5), as shown in Table 2; Figs. 4 and 5.

**Table 2:** Comparison of immunohistochemical markers between smokers and controls

Parameters	Control group *n* = 16 Median (IQR)	Smoker group *n* = 19 Median (IQR)	*P*-value
MPO positive cells/mm2	6 (4–8)	32 (21–39)	< 0.0001
EG2 positive cells/mm2	1.5 (0–3)	79 (68–95)	< 0.0001

The comparison was performed using Mann-Whitney test by SPSS 29. Data are presented as median (25–75% IQR), p-value < 0.05 was considered as statistically significant.

*EG2* eosinophilic granule, *IQR* interquartile range, *MPO* myeloperoxidase

### Correlation exists between certain laboratory biomarkers

**Fig. 6:**
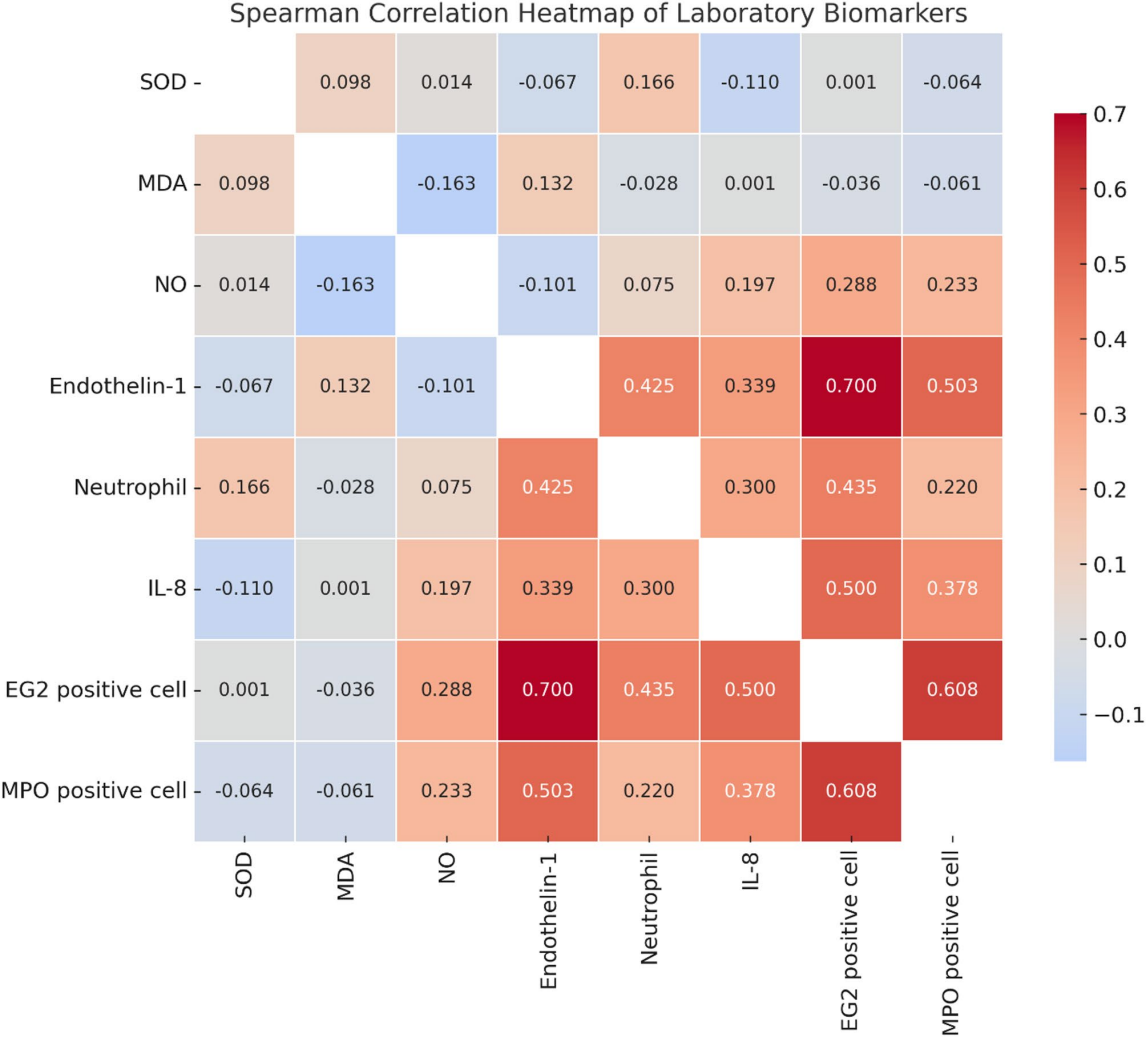
Spearman correlation heatmap among oxidative stress markers, inflammatory mediators, and granulocyte-related biomarkers in smokers. The figure illustrates the strength and direction of correlations between superoxide dismutase (SOD), malondialdehyde (MDA), nitric oxide (NO), endothelin-1, neutrophils, interleukin-8 (IL-8), EG2-positive eosinophils, and myeloperoxidase (MPO)-positive cells. Strong positive correlations were observed between EG2-positive cells and endothelin-1, IL-8, and MPO-positive cells, highlighting a potential interplay between oxidative stress and eosinophilic inflammation in smoking-associated pathophysiology. Significant correlations (*p* < 0.05) are indicated in the figure

Significant positive relationships are observed among IL-8, endothelin-1, MPO positive cells, and EG2 positive cells, as revealed by the correlation analysis. Significant positive correlations were found between IL-8 and endothelin-1 (*r* = 0.339, *p* < 0.0001), MPO positive cells (*r* = 0.378, *p* < 0.0001), and EG2 positive cells (*r* = 0.500, *p* < 0.0001). There is a significant correlation between endothelin-1 and MPO positive cells (*r* = 0.503, *p* < 0.0001), as well as EG2 positive cells (*r* = 0.700, *p* < 0.0001). MPO-positive cells and EG2-positive cells exhibit a notable positive correlation (*r* = 0.608, *p* < 0.0001), as indicated in Table 3; Fig. 6.

**Table 3:** Spearman correlation between certain laboratory biomarkers

		SOD	MDA	NO	Endothelin-1	Neutrophil	IL-8	EG2 positive cell	MPO positive cell
SOD	r		0.098	0.014	− 0.067	0.166	− 0.110	0.001	− 0.064
	p-value	.	0.172	0.850	0.351	0.021	0.126	0.992	0.376
MDA	r	0.098	1.000	− 0.163^*^	0.132	− 0.028	0.001	− 0.036	− 0.061
	p-value	0.172	.	0.023	0.067	0.696	0.984	0.617	0.394
NO	r	0.014	− 0.163		− 0.101	0.075	0.197	0.288	0.233
	p-value	0.850	0.023	.	0.060	0.299	0.006	< 0.0001	0.001
Endothelin-1	r	− 0.067	0.132	− 0.101		0.425	0.339	0.700	0.503
	p-value	0.351	0.067	0.060	.	< 0.0001	< 0.0001	< 0.0001	< 0.0001
Neutrophil	r	0.166	− 0.028	0.075	0.425		0.300	0.435	0.220
	p-value	0.021	0.696	0.299	< 0.0001	.	< 0.0001	< 0.0001	0.002
IL-8	r	− 0.110	0.001	0.197	0.339	0.300		0.500	0.378
	p-value	0.126	0.984	0.006	< 0.0001	< 0.0001	.	< 0.0001	< 0.0001
EG2 positive cell	r	0.001	− 0.036	0.288	0.700	0.435	0.500		0.608
	p-value	0.992	0.617	< 0.0001	< 0.0001	< 0.0001	< 0.0001	.	< 0.0001
MPO positive cell	r	− 0.064	− 0.061	0.233	0.503	0.220	0.378	0.608	
	p-value	0.376	0.394	0.001	< 0.0001	0.002	< 0.0001	< 0.0001	.

The correlation was done via Spearman test. A p-value below 0.05 is considered statistically significant

*EG2* eosinophilic granule, *IL* interleukin, *MDA* malondialdehyde, *MPO* myeloperoxidase, *NO* nitric oxide, *SOD* superoxide dismutase activity

## Discussion

This study aimed to explore the impact of heavy smoking on hematological parameters, oxidative stress parameters, and endothelin-1 in the Erbil Kurdistan region. The findings provide significant insight into the potential consequences of smoking on several physiological parameters. The data reveal that heavy cigarette users had unique changes in key laboratory indicators compared to the control group, which may have health implications. Notably, there was no significant difference in age and BMI between the control group and smokers. This homogeneity enhances the accuracy and reliability of our results by minimizing confounding variables related to age and BMI. Furthermore, the inclusion of only male participants eliminates the potential confounding effects of hormonal variations and gender disparities, thereby ensuring that the observed effects are primarily attributable to smoking, rather than gender-related differences.

The analysis reveals significant differences in several hematological parameters between smokers and healthy controls. Smoking is associated with increased RBC, Hb, WBC, and neutrophil counts and decreased platelet counts. These results are consistent with prior research that suggests smoking is linked to higher red cell mass and hemoglobin levels caused by carbon monoxide in cigarette smoke, inducing hypoxia—besides nicotine, which promotes vasoconstriction and reduces oxygen delivery. Hypoxia triggers erythropoiesis, causing an increase in RBC and Hb levels. In addition, the elevated neutrophil count in smokers suggests an enhanced systemic inflammatory response, which may play a role in the early stages of vascular dysfunction and chronic inflammatory conditions associated with smoking, such as cardiovascular and respiratory diseases^22^.

Nicotine induces oxidative stress by activating nicotinamide adenine dinucleotide phosphate (NADPH) oxidase and reducing antioxidant defenses, contributing to cellular damage and inflammation^23^. Additionally, carbon monoxide—a toxic gas produced during the combustion of tobacco—binds to hemoglobin with an affinity approximately 200–250 times greater than oxygen, forming carboxyhemoglobin (COHb), which impairs oxygen delivery to tissues and induces tissue hypoxia^24^. Chronic hypoxia can trigger compensatory erythropoiesis, oxidative stress, and alterations in immune function. These mechanisms may collectively explain some of the smokers’ biochemical and hematological alterations.

Increased WBC counts in smokers may reflect an inflammatory response to the toxic components of cigarette smoke, which can induce a chronic inflammatory state. Neutrophilia, or elevated neutrophil counts, is also commonly observed in smokers due to this inflammatory response (Moriarty et al., 2021). The effect of smoking on platelet count is still controversial^25^. The reduced platelet counts observed in smokers in the current study could be attributed to the toxic effects of smoking on bone marrow function or platelet survival. Although previous studies have reported that cigarette smoking is associated with elevated platelet counts due to increased bone marrow stimulation and systemic inflammation^26–28^, the current study presents an opposing trend with lower platelet counts observed among smokers. This discrepancy may be attributed to several factors. First, chronic exposure to tobacco smoke can induce bone marrow suppression in some individuals, particularly at high levels of exposure^29^, thereby reducing platelet production. Second, oxidative stress and endothelial dysfunction caused by prolonged smoking may accelerate platelet activation and consumption, effectively decreasing the circulating platelet count^27,30^. Lastly, differences in population characteristics, such as nutritional status, comorbid conditions, or even genetic polymorphisms affecting hematopoiesis, may account for this variation. These factors should be considered when interpreting divergent findings in platelet parameters among smokers (Hoffman et al., 2018).

The neutrophil count in heavy cigarette smokers was significantly higher than in the control group. Neutrophils are white blood cells that have an important role in fighting infection and inflammation. Elevated neutrophil counts suggest an ongoing inflammatory response, possibly contributing to the development of smoking-related diseases such as chronic obstructive pulmonary disease (COPD) and cardiovascular disorders. Our findings are consistent with previous studies, such as those by Elisia, et al.^31^, who also observed increased neutrophil counts in smokers and associated with poor survival outcomes, highlighting the important prognostic significance of neutrophil-mediated inflammation in smoking-related health risks.

However, there was no statistical difference in lymphocyte counting between cigarette smokers and the control group. Lymphocytes are immune cells that play an important role in immune defense and can be affected by different factors, including smoking. On the other hand, it is important to note that while our findings did not show an effect of smoking on lymphocyte count, smoking may still influence the immune system through other complex immunological pathways^32^. This discrepancy highlights the complex and selective nature of smoking-induced hematological changes.

Although previous studies have reported that smoking can transiently elevate blood pressure due to nicotine-induced catecholamine release and vasoconstriction^33^, our findings did not demonstrate a significant increase in blood pressure among the smokers in our study. One possible explanation is that chronic exposure to nicotine may lead to the development of tolerance to its pressor effects over time, particularly in habitual or heavy smokers, resulting in a blunted cardiovascular response^34^. Moreover, genetic, environmental, and racial differences may influence individual susceptibility to smoking-induced hemodynamic changes. For instance, it has been suggested that certain populations exhibit greater vascular adaptation or compensatory mechanisms in response to chronic smoking, potentially mitigating blood pressure elevation^35^.

Heavy smokers exhibit elevated levels of MDA due to exposure to cigarette smoke, which contains this α,β-unsaturated aldehyde^36^. Heavy smokers exhibit elevated levels of MDA, a key biomarker of lipid peroxidation, due to chronic exposure to reactive compounds in cigarette smoke. Among these, α, β-unsaturated aldehydes, such as acrolein, play a critical role. These highly electrophilic aldehydes react with cellular lipids, initiating and propagating oxidative damage. Acrolein, in particular, has been shown to enhance the formation of MDA by disrupting antioxidant defenses and promoting the oxidation of polyunsaturated fatty acids. The increased presence of acrolein in cigarette smoke^37^ directly contributes to higher systemic MDA levels in heavy smokers, indicating enhanced oxidative stress^38^. The positive correlation between MDA level and the number of cigarettes smoked per day was recorded by^39^. The toxic effects of MDA on human lung fibroblasts have been demonstrated, leading to reduced cell proliferation, altered morphology, increased apoptosis, and DNA damage, highlighting the impact of MDA on cellular and DNA levels in heavy smokers^40^.

The study further assessed the concentrations of NO in heavy smokers and healthy control groups. As observed in our study, elevated NO levels in heavy smokers indicate increased production of reactive oxygen species, which exuberate oxidative damage. This finding aligns with^41^. Conversely, research indicates that heavy smokers exhibit lower NO levels in comparison to nonsmokers^42^. Cigarette smoke contains free radicals that contribute to oxidative stress-related diseases, with NO reacting with hydrogen peroxide to generate cytotoxic singlet oxygen^43^. Additionally, depending on our result, SOD level is lower in heavy cigarette smokers compared to the control group. This finding is supported by ^44^ and ^45^, both recorded that cigarette smokers had significantly lower SOD levels compared to the control, highlighting the impact of cigarette consumption on antioxidant enzyme activity and oxidative stress levels in the body.

Compared to healthy controls, the research studies provide valuable insights into the levels of IL-8 in smokers. The studies conducted by Sahibzada, et al.^46^ and Zhang and Bai^47^ reveal a clear correlation between smoking and elevated levels of IL-8, surpassing those found in nonsmokers or healthy individuals. Moreover, the research conducted by Nanakaly, et al.^48^ emphasizes that smokers with periodontitis exhibit elevated levels of salivary IL-8 compared to nonsmokers with periodontitis and healthy controls. The collective findings point to a consistent increase in IL-8 levels among smokers, suggesting a potential link between smoking and inflammatory reactions. Neutrophils are attracted to sites of inflammation by IL-8, a highly potent pro-inflammatory chemokine. Smokers who are chronically exposed to tobacco smoke experience ongoing inflammation in their airways and body, resulting in higher levels of IL-8. As a result, the bone marrow is stimulated to generate and release additional WBCs to fight the persistent inflammation^14^.

This research also showed the higher levels of MPO-positive cells in bronchial biopsies of smokers compared with the healthy control, further highlighting the involvement of inflammatory processes in individuals with heavy smoking. The lung’s pathophysiology involves MPO-positive cells, such as neutrophils and monocytes. Neutrophils, being cytotoxic, can lead to tissue damage^49^. The study demonstrated elevated levels of EG2 (activated eosinophil) in smokers compared to healthy individuals. In smokers, eosinophil mediators, such as ECP, play a significant role in the pathology by inducing inflammation. The assessment in this study also focuses on measuring antibodies to ECP (EG2 type), which indicates eosinophil activation^49^. EG2-positive activated eosinophils are key contributors to the development of smoking-related lung inflammation and remodeling^50^. In addition, eosinophils that have been activated, which are distinguished by their ability to secrete inflammatory substances, have a role in causing local inflammation and tissue damage, particularly through the release of toxic proteins and lipid mediators^51^. Cigarette smoke components activate eosinophils, causing the release of inflammatory mediators such as IL-8 and IL-6, worsening lung inflammation and possibly triggering airway hyperresponsiveness^50^.

One of the most notable findings of our study is a significant elevation of endothelin-1 in heavy smokers, showcasing its detrimental effects on various organs. Research highlights that cigarette smoke exposure induces up-regulation of endothelin receptors, mediating vascular and airway hyper-reactivity. This can result in abnormal contraction and adverse proliferation in the vasculature and airways^52^. Heavy smoking increases endothelin-1 levels and expression through different mechanisms. Firstly, nicotine from smoking stimulates ET-1 production, contributing to vasoconstriction and tissue hypoxemia^53^.

Additionally, smoking induces the release of endothelin-1 into blood vessels, causing vasoconstriction and subsequent ischemia in organs and leading to structural changes and dysfunction^53^. Moreover, experimental studies have demonstrated nicotine’s influence on endothelin-1 synthesis and receptor expression, highlighting the stimulatory effect of nicotine on endothelin-1 production^11^. Also, young smokers reveal higher pulmonary extraction of endothelin-1 and more pronounced renal vasoconstriction^54^. Our data suggest that endothelin-1 plays a crucial role in smoking-induced vascular dysfunction, supporting^55^ but also providing new insights into the extent of endothelin-1’s involvement.

The significant positive associations found among IL-8, endothelin-1, MPO positive cells, and EG2 positive cells are due to their functions in inflammatory and immune responses. Neutrophils and other immune cells are recruited and stimulated by IL-8, a pro-inflammatory cytokine, to the sites of inflammation^56^. The involvement of endothelin-1, a powerful vasoconstrictor, in inflammation can be due to its upregulation of IL-8 ^57^. During inflammation, MPO positive cells, including neutrophils, release MPO as part of the oxidative burst^58^. During smoking, activated eosinophils (EG2 positive cells) play a vital role in allergic reactions and show a significant correlation with IL-8, as this cytokine attracts eosinophils to the site of inflammation^58^. The interaction of these markers suggests a coordinated reaction to inflammation and immune activation, where one marker’s presence or activity can influence or reflect the activity of others.

This study has several strengths. It is among the few original studies in the region to combine hematological, biochemical, and histological data to evaluate the systemic effects of heavy smoking. Including serum markers and bronchial biopsy analysis provides a comprehensive view of oxidative stress and inflammation. Additionally, matching participants for age and BMI helped minimize the impact of confounding factors.

However, the study also has limitations. The cross-sectional design prevents causal inference, and the exclusion of female participants limits generalizability to the broader population. However, due to cultural and societal norms in our region, female smoking is generally stigmatized, and very few women openly report smoking. As a result, recruiting female smokers was not possible. The relatively small sample size for biopsy analysis may not capture the full histological variation among smokers. Finally, the absence of long-term follow-up data restricts our ability to assess the progression or reversibility of the observed changes.

## Conclusion

The significantly elevated levels of oxidative stress markers, inflammatory cytokines, and vascular dysfunction indicators among heavy smokers highlight the urgent need for clinical and public health interventions. These biomarkers—such as IL-8, endothelin-1, MPO, and MDA—may serve as early indicators of systemic damage and support risk stratification in clinical settings. Public health efforts should prioritize the monitoring of such biomarkers and expand smoking cessation programs to reduce the burden of smoking-related diseases and promote long-term vascular and respiratory health.

## Data Availability

The datasets generated during and/or analyzed during the current study are not publicly available due to data will be used for analyzing again for publication but are available from the corresponding author upon reasonable request.
